# Disparities in Receipt of National Comprehensive Cancer Network Guideline-Adherent Care and Outcomes among Women with Triple-Negative Breast Cancer by Race/Ethnicity, Socioeconomic Status, and Insurance Type

**DOI:** 10.3390/cancers15235586

**Published:** 2023-11-26

**Authors:** Chimezie D. Ubbaonu, Jenny Chang, Argyrios Ziogas, Rita S. Mehta, Kari J. Kansal, Jason A. Zell

**Affiliations:** 1 Division of Hematology/Oncology, Department of Medicine, School of Medicine, University of California Irvine, Orange, CA 92868, USA; rsmehta@hs.uci.edu (R.S.M.); jzell@hs.uci.edu (J.A.Z.); 2 Department of Internal Medicine, University of California, Irvine, CA 92868, USA; jjchang@hs.uci.edu (J.C.); aziogas@hs.uci.edu (A.Z.); 3 Chao Family Comprehensive Cancer Center, University of California Irvine Medical Center, Orange, CA 92868, USA; kkansal@hs.uci.edu; 4 Division of Surgical Oncology, Department of Surgery, University of California Irvine Medical Center, Orange, CA 92868, USA

**Keywords:** disparities, outcomes, NCCN, breast cancer, TNBC

## Abstract

**Simple Summary:**

Breast cancer is the most diagnosed cancer in women. Women who are diagnosed early and who receive evidence-based guideline-adherent care have better outcomes. Previous studies have shown disparities in the receipt of appropriate care based on race and socioeconomic status. This study was designed to explore these disparities in women diagnosed with triple-negative breast cancer in the context of the receipt of National Comprehensive Cancer Network guideline-adherent care and its effects on outcomes using the California Cancer Registry. Our results show that racial minorities and members of lower socioeconomic groups are less likely to receive guideline-adherent care, and that this is associated with an increased risk of dying from breast cancer. Our study adds to the growing evidence of persistent healthcare disparities, suggesting that more work is needed to bridge the gaps in healthcare provision for racial minorities and members of lower socioeconomic groups.

**Abstract:**

Background: The National Comprehensive Cancer Network guidelines were designed to improve patient outcomes. Here, we examine factors that may contribute to outcomes and guideline adherence in patients with triple-negative breast cancer. Methods: This was a retrospective cohort study of women with triple-negative breast cancer using the California Cancer Registry. Adherent treatment was defined as the receipt of a combination of surgery, lymph node assessment, adjuvant radiation, and/or chemotherapy. A multivariable logistic regression was used to determine the effects of independent variables on adherence to the NCCN guidelines. Disease-specific survival was calculated using Cox regression analysis. Results: A total of 16,858 women were analyzed. Black and Hispanic patients were less likely to receive guideline-adherent care (OR 0.82, 95%CI 0.73–0.92 and OR 0.87, 95%CI 0.79–0.95, respectively) compared to White patients. Hazard ratios adjusted for adherent care showed that Black patients had increased disease-specific mortality (HR 1.28, 95%CI 1.16–1.42, *p* < 0.0001) compared to White patients. Conclusions: A significant majority of breast cancer patients in California continue to receive non-guideline-adherent care. Non-Hispanic Black patients and patients from lower SES quintile groups were less likely to receive guideline-adherent care. Patients with non-adherent care had worse disease-specific survival compared to recipients of NCCN guideline-adherent care.

## 1. Introduction

Breast cancer remains the most commonly diagnosed cancer and the second-leading cause of cancer mortality in American women [1,2]. The lifetime risk of developing invasive breast cancer is 13%, and this translates into an estimated 297,790 new cases in 2023 [3]. Although, on average, 10% of women diagnosed with breast cancer are estrogen receptor (ER)-negative, progesterone receptor (PR)-negative, and human epidermal growth factor receptor 2 (HER2)-negative (i.e., triple-negative breast cancer; TNBC), this proportion increases to 19% of cases in Non-Hispanic Black women [1].

Triple-negative breast cancer is important because limited targeted therapies are currently available for its treatment. TNBC tends to be more aggressive and presents at an earlier age with more advanced disease. As such, it carries a worse short-term prognosis compared to receptor-positive breast cancers, and even more so when detected later in the disease course [4]. Despite the aggressiveness of TNBC, women diagnosed with early, node-negative tumors <1 cm (T1a, bN0M0) have been shown to have an excellent prognosis, with a distant relapse-free survival at 5.5 years of more than 90% [5]. The early detection of breast cancer (and, by extension, triple-negative breast cancer) using mammography can significantly improve outcomes in these women. This is especially true for Non-Hispanic Black women, who are disproportionately affected by TNBC [1].

Previous studies have shown that disparities in access to healthcare among Non-Hispanic Black and Hispanic women continue to be a barrier to receiving guideline-adherent care (GAC) [6]. Adherence to evidence-based management improves outcomes in patients with breast cancer [7,8]. A few studies have looked at disparities in treatment and outcomes in triple-negative breast cancer patients [9,10]. However, the available studies did not design a treatment algorithm based on GAC by cancer stage. Our study aims to fill this gap by exploring the impact of socioeconomic factors on the receipt of GAC in women diagnosed with TNBC.

## 2. Methods

This retrospective cohort study of adult women diagnosed with triple-negative breast cancer (TNBC) in California was carried out using data from the California Cancer Registry (CCR). Patients diagnosed between 2004 and 2015 (with follow-up through November 2018) were included in the study. This study was approved by the Institutional Review Board of the University of California, Irvine, as exempt.

### 2.1. Data Source

The CCR is California’s population-based cancer surveillance system, which collects information about almost all cancers diagnosed and treated in the state. Data include patient-level demographics, tumor-specific characteristics (including information on the timing and receipt of chemotherapy, surgery, and radiation therapy), and vital statistics such as time and cause of death. Treatment data obtained from the registry are limited to the first course of treatment.

**Figure 1:**
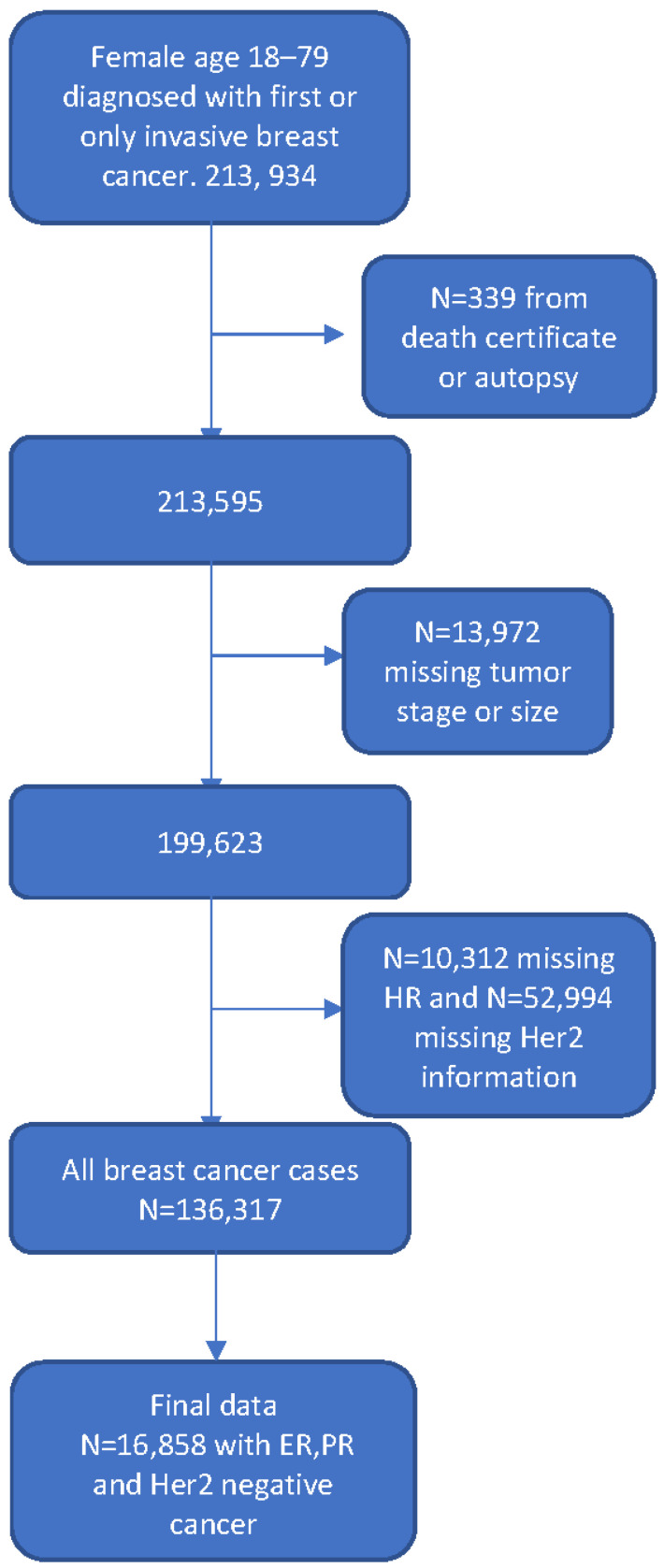
Flow chart showing steps in sample exclusion. Abbreviations: ER, estrogen receptor; PR, progesterone receptor; HER2, human epidermal growth factor receptor 2.

The inclusion criteria for the study were female patients who were 18 to 79 years old and diagnosed with triple-negative invasive carcinoma of the breast as the first or only cancer in their lifetime. Patients older than 80 years were excluded because comorbidity data were missing, and we believe that this may have influenced the decision to withhold guideline-recommended therapy. TNBC is defined as breast cancer that is estrogen receptor (ER)-negative, progesterone receptor (PR)-negative, and human epidermal growth factor receptor 2 (HER2)-negative. Figure 1 shows the steps taken during sample exclusion. The final analytic data included 16,858 TNBC cases.

The following variables were collected for this study: age at diagnosis, year of diagnosis, race/ethnicity (Non-Hispanic White (NHW), Non-Hispanic Black (NHB), Asian (not including Pacific Islanders), and other or unknown), mutually exclusive insurance type (managed care, Medicare, Medicaid, uninsured, other insurance/unknown), marital status (married vs. not married), neighborhood socioeconomic status (nSES), tumor stage and grade, and treatment variables (surgery, chemotherapy, radiation, and hormonal therapy). Neighborhood socioeconomic status (nSES) was determined using neighborhood census block data and was divided into five groups by quintiles (lowest SES, lower-middle SES, middle SES, higher-middle SES, and highest SES) based on the Yost SES score or Yang SES index [11]. The Yost score and Yang index are composite indexes of census block group-level variables used to determine socioeconomic status based on, amongst others, income, median home value, educational attainment, and occupation [11].

An algorithm based on the NCCN-recommended treatment approach for TNBC was applied according to the time of diagnosis (2004–2015) and is summarized in supplemental Table S1 [12,13,14,15]. The algorithmic steps from the NCCN guidelines were unchanged during the study period. A patient was defined as having received NCCN guideline-adherent care if they received any combination of surgery, radiation, or chemotherapy appropriate for the nodal status and American Joint Committee on Cancer (AJCC) stage as follows [16]:

- Patients with localized disease and up to 1–3 positive lymph nodes (stages I, II, IIIA + N1) with node-negative tumors ≤ 10 mm who received sentinel lymph node surgery and either a total mastectomy or breast-conserving surgery (BCS) plus radiation.
- Patients with localized disease and up to 1–3 positive lymph nodes (stages I, II, IIIA + N1) with node-negative tumors > 10 mm who underwent sentinel lymph node surgery, chemotherapy, and either a total mastectomy or BCS plus radiation.
- Patients with localized disease and up to 1–3 positive lymph nodes (stages I, II, IIIA + N1) with nodal micro-metastasis, tumor ≤ 10 mm, who received sentinel lymph node surgery and either a total mastectomy or BCS plus radiation.
- Patients with localized disease and up to 1–3 positive lymph nodes (stages I, II, IIIA + N1) with nodal micro-metastasis, tumor > 10 mm, who received sentinel lymph node surgery, chemotherapy and either a total mastectomy or BCS plus radiation.
- Patients with localized disease and up to 1–3 positive lymph nodes (stages I, II, IIIA + N1) with lymph-node-positive tumors of any size who received sentinel lymph node surgery and chemotherapy.
- Patients with stages IIIA, IIIB, or IIIC tumors who received sentinel lymph node surgery and chemotherapy.
- Patients with stage IV cancer who received chemotherapy.

A subject was considered to have received adherent care if treatment was consistent with NCCN treatment guidelines for the tumor stage [16]. Based on this, the treatment was dichotomized into adherent vs. non-adherent groups. The primary purpose of the study was to examine the correlation between race/ethnicity, nSES, and insurance type and the receipt of NCCN guideline-adherent care. The secondary purpose was to investigate the effect of NCCN guideline-adherent care on breast-cancer-specific survival. Breast-cancer-specific survival was defined as the time from diagnosis of breast cancer to death due to breast cancer. Patients were censored if they were alive until the end of the follow-up or if they had died from other causes.

### 2.2. Statistical Analysis

Descriptive statistics of the study population according to treatment adherence status were generated for the patients’ social–demographic variables and tumor characteristics. The chi-squared test was used to test for differences between the guideline-adherent and guideline-non-adherent groups. Univariate and multivariable logistic regression was fitted for the receipt of NCCN guideline-adherent care. NCCN guideline-adherent care was the dependent variable in this analysis. Independent variables were age, race, nSES, insurance type, marital status, and tumor characteristics. We conducted survival analyses using disease-specific survival. Patients who died from other causes or were alive at the end of the follow-up were censored. Kaplan–Meier estimates of survival probability and log-rank tests were performed to compare adherent and non-adherent groups. After checking proportional hazards assumptions, univariate and multivariate Cox proportional hazards regression models were fitted for disease-specific survival. Unadjusted and adjusted hazard ratios with 95% confidence intervals were calculated. Statistical significance was set at *p* < 0.05 using 2-tailed tests. Statistical analyses were performed using SAS 9.4 (SAS Institute Cary, NC, USA).

## 3. Results

Patient demographics are summarized in Table 1. The distribution of patient characteristics by race/ethnicity can be found in supplemental Table S2. A total of 16,858 women who were diagnosed between 2004 and 2015 were included in the study analysis. Of these, 5472, or 32.5%, received NCCN guideline-adherent care. The age group 18–44 had the lowest proportion of cases (*n* = 3533 or 21.0%, *p* < 0.0001), while the age group 65+ was least likely to receive adherent care (*n* = 1198 or 28.8%, *p* < 0.0001) compared to other age groups (Table 1).

When compared to Non-Hispanic White patients (34.0%), Non-Hispanic Black patients were the least likely to receive guideline-adherent care (29.0%, *p* < 0.0001). A similar association was seen among uninsured women (30.3%, *p* < 0.0001), who were less likely to receive guideline-adherent care than women having managed care insurance (33.5%). Women in the lowest SES group (27.9%, *p* < 0.0001) were least likely to receive guideline-adherent care compared to women with the highest SES (34.9%).

Adjusted odds ratios for receiving NCCN guideline-adherent care are reported in Table 2. When compared to Non-Hispanic White women, after adjusting for SES and insurance type, Non-Hispanic Black and Hispanic women were significantly less likely to receive guideline-adherent care (respectively, OR, 0.82, 95% CI 0.73–0.92, and OR, 0.87, 95% CI 0.79–0.95). Socioeconomic status had a strong effect on the likelihood of receiving guideline-adherent care, with the lowest SES and lower-middle SES least likely to receive adherent care when compared to those in the highest SES as a referent group (respectively, OR, 0.77 [95% CI 0.68–0.87], and OR, 0.88 [95% CI 0.79–0.98]; *p* < 0.0001). Married women were more likely to receive guideline-adherent care compared to women who were not married (OR, 1.17 [95% CI 1.09–1.26]; *p* < 0.0001). When compared to women with stage I cancer, those with stage IV cancer were the most likely to receive guideline-adherent care (OR, 7.47 [95% CI 6.04–9.24]; *p* < 0.0001). Compared to women who had managed care insurance, having Medicare (OR, 0.92 [95% CI 0.83–1.03]; *p* = 0.16), Medicaid (OR 0.97 [95% CI 0.86–1.09]; *p* = 0.57), other insurance (OR, 0.97 [95% CI 0.88–1.07]; *p=* 0.54), or no insurance (OR, 0.90 [95% CI 0.74–1.09]; *p* =0.27) had no significant effect on the likelihood of receiving guideline-adherent care (Table 2).

The hazard ratios for breast-cancer-specific death according to age, race/ethnicity, insurance type, SES, marital status, tumor stage, and the receipt of NCCN guideline-adherent care using a Cox model are listed in Table 3. In multivariable analysis, when compared to Non-Hispanic White women as a reference group, Non-Hispanic Black women were more likely to die from TNBC (HR, 1.28 [95% CI 1.16–1.42]; *p* < 0.0001) (Kaplan–Meier survival curves can be found in supplemental Figure S1). Women who had Medicare or Medicaid insurance were also more likely to die when compared to those with managed care insurance (respectively, HR, 1.20 [95% CI 1.08–1.34]; *p* = 0.001; HR, 1.29 [95% CI 1.16–1.43]; *p* < 0.0001). There was a trend toward statistically significantly higher mortality in uninsured women (HR, 1.17 [95% CI 0.98–1.40]; *p* = 0.08). The SES of women had a very strong effect on the risk of death from breast cancer. This effect was seen in the lowest SES (HR, 1.20 [95% CI 1.06–1.36]; *p* = 0.004), lower-middle SES (HR, 1.19 [95% CI 1.06–1.33]; *p* = 0.004), and middle SES (HR, 1.21 [95% CI 1.08–1.35]; *p* = 0.0001) groups. Not surprisingly, having more advanced disease was associated with an increased risk of death, with the highest risk seen in stage IV tumors (HR, 37.82 [95% CI 32.74–43.68]; *p* < 0.0001).

## 4. Discussion

This study reveals the persistence of significant disparities in the treatment and outcomes of breast cancer among U.S. women, as described in previous studies [17,18,19,20]. Our study specifically demonstrates a racial difference in the odds of receiving National Comprehensive Cancer Network (NCCN) guideline-adherent care for women with triple-negative breast cancer (TNBC). We adjusted for patients’ age, neighborhood socioeconomic status, insurance type, and tumor stage and observed that significant differences persisted in the odds of receiving guideline-adherent care. We also observed increased breast cancer-specific mortality for NHB women compared to NHW women. After adjusting for neighborhood socioeconomic status and insurance type, the magnitude of the difference was reduced but remained statistically significant.

Age has been recognized as an independent risk factor for poor health outcomes. As people get older, they develop cardiovascular, metabolic, and other comorbidities and physical frailty, which independently contribute to increased mortality risk [21,22]. Other factors, such as impaired mobility, cognitive impairment, loneliness, and lack of social support, are associated with advancing age. All of these factors have been shown to impact health outcomes in the elderly. By adjusting for age, we aimed to reduce or eliminate any confounding effects of this variable. In this study, age had minimal effects on the odds of receiving NCCN guideline-adherent care. There was also no significant difference in mortality risk in either the unadjusted or adjusted analysis. This was despite the NHW population having more subjects in the 65–79-year age range (30.1%) compared to NHB (22.6%). The NHB population of note also had more subjects in the young (18y–44y) age range, who typically tend to have more aggressive disease at diagnosis, which is associated with poorer outcomes. Although admittedly, in this study, we did not carry out a subgroup analysis to further explore associations, our observation suggests that age by itself does not independently increase the risk of having poorer outcomes in the presence of proper access to quality healthcare.

The effect of the cancer stage at diagnosis on the likelihood of receiving NCCN guideline-adherent care was surprisingly strong in this study. When compared to women with stage I disease, stage IV disease patients were 7 times more likely to receive NCCN guideline-adherent care. Prior studies have shown that patients with no insurance or who have public insurance such as Medi-Cal tended to present at later stages of cancer compared to those with private insurance [23]. Despite this, stage IV cancer patients were more likely to receive guideline-adherent care, probably because the management of early-stage disease requires a multidisciplinary approach (including breast surgeons, medical oncologists, and radiation oncologists), whereas stage IV disease patients are most commonly treated with palliative chemotherapy. Since NHB women were more likely to have advanced disease at diagnosis, it is no surprise that they had worse outcomes.

Socioeconomic status (SES) is an important contributing factor to health outcomes in populations. Measures of SES can be at the level of the individual or their neighborhood [24]. Previous studies have shown that nSES influences health outcomes in individuals independent of the individual SES [25]. Our analysis using nSES showed that 27.9% of women in the lowest nSES received guideline-adherent care, compared to 35% in the highest nSES. This translated to a 23% chance of not receiving guideline-adherent care compared to the highest nSES after adjusting for race and insurance status.

In the U.S., the African American population is disproportionately poor, undereducated, unemployed, and more likely to live in poorer neighborhoods. Between 2014 and 2016, the average household income in the U.S. was USD 70,000 compared to USD 48,000 for African Americans [26,27]. Lower income usually equates to living in poorer neighborhoods with higher levels of crime, environmental pollution, and less access to sources of healthy food options due to cost [28,29]. Furthermore, because lower levels of education and skill acquisition correlate with higher unemployment rates, patients with low SES are less likely to have private insurance, which is typically linked to employment status.

The health insurance type revealed a significant correlation with health outcomes. Women with Medicaid insurance had a 29% increased risk of death compared to managed care recipients after adjusting for age, nSES, and disease stage. When we looked at the risk of death in NHB compared to NHW women, we adjusted for insurance type but observed a residual disparity. This is likely because having health insurance does not always equate to having access to good-quality healthcare. The residual disparity suggests that other factors are likely at play. These factors may include a lack of reliable transportation to appointments, language barriers, and difficulty navigating the healthcare system due to limited education [30]. For example, the Affordable Care Act (ACA) significantly reduced gaps in healthcare coverage and access to mammography services among Hispanics and African Americans between 2008 and 2015, although it currently remains unclear whether this has translated into a reduction in disparate breast cancer outcomes [31].

Like our study, other authors have observed a residual disparity in disease outcomes in NHB women compared to NHW women, and differences in tumor biology have been implicated as contributory, independent of SES and access to healthcare [32]. Breast cancers in NHB women are more likely to harbor deleterious mutations such as p53 and have higher histologic and nuclear grades than those in NHW women [33,34,35]. Inflammatory breast cancer is a relatively rare and aggressive type of breast cancer, which presents at an earlier age and has also been observed to disproportionately affect Non-Hispanic Black women compared to NHW women [36]. Epigenetic differences have also been described between these groups. Mehrotra and colleagues reported significant differences between NHB and NHW women in the methylation of HIN-1 (regulation of epithelial cell proliferation and differentiation), Twist (apoptosis regulation), cyclin D2 (cell cycle regulation), RAR-β (inhibition of proliferation), and RASSF1A (putative tumor suppressor gene) [37]. This association was greatest among women <50 years old with ER/PR-negative breast cancer.

Our study, being a retrospective population-based cohort study, has several limitations. We relied on registry data from the California Cancer Registry. California is the most racially diverse state in the U.S. [38] and represents a geographically contiguous region uniquely suited for cancer health disparity research. However, the generalization of the findings to the entire country must be performed with caution. In California, most women are eligible for Medi-Cal (the state’s equivalent of Medicaid) when diagnosed with breast cancer. This means that the proportion of women with no insurance prior to diagnosis may have been underestimated. Furthermore, insurance status, as captured by the registry, may not coincide with the time of cancer diagnosis. Research has shown that neighborhood SES appears to have similar but independent effects on the health of the individual [24,25]. We estimated the socioeconomic status of women using neighborhood-level census block data, which may not accurately reflect individual-level socioeconomic status, nor would it necessarily fully capture the effects on our study outcomes. The validity of the treatment data used in our analysis is dependent on how much of the treatment data were captured by the cancer registry. In this study, treatment data were available for up to 6 months post-diagnosis. Cancer outcomes depend on the quality of care received. The quality of surgery, the duration of radiation therapy, and the number of cycles of chemotherapy could not be ascertained from the available data. Finally, there may be interactions between socioeconomic, insurance, and other unmeasured variables, such as comorbidities unaccounted for in our multivariate regression models.

In conclusion, we observed that race/ethnic disparities in the receipt of NCCN guideline-adherent care and breast-cancer-specific mortality were attenuated but persisted after adjustment for socioeconomic status and insurance type. The demonstrated attenuation in the risk of adverse outcomes between NHB and NHW women by correcting for nSES and insurance type, even if not complete, demonstrates the need for concerted efforts aimed at improving socioeconomic conditions for NHB women; health policy changes that make health insurance available to all Americans; cultural sensitivity training within health systems to improve the quality of doctor–patient interactions; and funding for research into possible molecular/genetic bases for differences in tumor behavior.

## Figures and Tables

**Table 1:** Distribution of patient characteristics by the status of treatment.

	Total N	%	NCCN-Adherent N	%	NCCN-Non-Adherent N	%	*p*-Value ^a^
Total	16,858	100	5472	32.5	11,385	67.5	
Age at diagnosis (years)							<0.0001
18–44	3533	21	1161	32.9	2372	67.1	
45–54	4568	27	1526	33.4	3042	66.6	
55–64	4594	27.3	1587	34.5	3007	65.5	
65+	4163	24.7	1198	28.8	2965	71.2	
Year of diagnosis							<0.0001
2004–2009	10,193	60.5	3091	30.3	7102	69.7	
2010+	6665	39.5	2381	35.7	4284	64.3	
Race/ethnicity							<0.0001
Non-Hispanic White	8930	53.0	3037	34.0	5893	66.0	
Non-Hispanic Black	2085	12.4	605	29.0	1480	71.0	
Hispanic	3880	23.0	1175	30.3	2705	70.0	
Asian	1752	10.4	573	32.7	1179	67.3	
Other/unknown	211	1.3	82	38.9	129	61.1	
Insurance							<0.0001
Managed care	8610	51.1	2887	33.5	5723	66.5	
Medicare	2688	15.9	791	29.4	1897	70.6	
Medicaid	2093	12.4	645	30.8	1448	69.2	
Other insurance (FFS, Tricare, VA or NOS)	2884	17.1	971	33.7	1913	66.3	
Not insured or unknown	583	3.5	178	30.5	405	69.5	
Socioeconomic status (SES)							<0.0001
Lowest SES	2590	15.4	723	27.9	1867	72.1	
Lower-middle SES	3207	19.0	998	31.1	2209	68.9	
Middle SES	3501	20.8	1109	31.7	2392	68.3	
Higher-middle SES	3800	22.5	1331	35.0	2469	65.0	
Highest SES	3760	22.3	1311	34.9	2449	65.1	
Marital status							<0.0001
Married	9838	58.4	3348	34.0	6490	66.0	
Not married	7020	41.6	2124	30.3	4896	69.7	
Tumor stage							<0.0001
I	5737	34.0	2402	41.9	3335	58.1	
II	7800	46.3	1688	21.6	6112	78.4	
III	2645	15.7	819	31.0	1826	69.0	
IV	676	4.0	563	83.3	113	16.7	

Abbreviations: FFS, fee for service; VA, Veteran’s Affairs; NOS, not otherwise specified. ^a^ Chi-square test for the difference between non-adherent group and adherent group.

**Table 2:** Adjusted odds ratios from logistic regression on receiving NCCN-adherent care.

	Unadjusted OR (95% C.I.)	*p*-Value	Adjusted OR (95% C.I.)	*p*-Value
Age at diagnosis (years)	0.994 (0.991–0.997)	<0.0001	0.988 (0.984–0.991)	<0.0001
Race/ethnicity				
Non-Hispanic White	Ref		Ref	
Non-Hispanic Black	0.79 (0.72–0.88)	<0.0001	0.82 (0.73–0.92)	<0.001
Hispanic	0.84 (0.78–0.91)	<0.0001	0.87 (0.79–0.95)	0.003
Asian	0.94 (0.85–1.05)	0.292	0.92 (0.82–1.03)	0.14
Other/unknown	1.23 (0.93–1.63)	0.14	1.29 (0.96–1.73)	0.09
Insurance				
Managed care	Ref		Ref	
Medicare	0.83 (0.75–0.91)	<0.0001	0.92 (0.83–1.03)	0.16
Medicaid	0.88 (0.80–0.98)	0.02	0.97 (0.86–1.09)	0.57
Other insurance	1.01 (0.92–1.11)	0.89	0.97 (0.88–1.07)	0.54
Not insured or unknown	0.87 (0.73–1.05)	0.14	0.90 (0.74–1.09)	0.27
Socioeconomic status (SES)				
Highest SES	Ref		Ref	
Lowest SES	0.72 (0.65–0.81)	<0.0001	0.77 (0.68–0.87)	<0.0001
Lower-middle SES	0.84 (0.76–0.93)	0.0009	0.88 (0.79–0.98)	0.02
Middle SES	0.87 (0.79–0.96)	0.004	0.91 (0.82–1.01)	0.07
Higher-middle SES	1.01 (0.92–1.11)	0.88	1.03 (0.93–1.13)	0.61
Marital status				
Not married	Ref		Ref	
Married	1.19 (1.11–1.27)	<0.0001	1.17 (1.09–1.26)	<0.0001
Tumor stage				
I	Ref		Ref	
II	0.38 (0.36–0.41)	<0.0001	0.37 (0.34–0.40)	<0.0001
III	0.62 (0.57–0.69)	<0.0001	0.61 (0.55–0.67)	<0.0001
IV	6.92 (5.61–8.52)	<0.0001	7.47 (6.04–9.24)	<0.0001

**Table 3:** Adjusted and unadjusted hazard ratios for disease-specific mortality.

	Unadjusted HR (95% CI)	*p*-Value	Adjusted HR (95% C.I.)	*p*-Value
Age at diagnosis (years)	0.997 (0.994–1.0)	0.064	1.003 (1.0–1.006)	0.06
Year of Diagnosis	1.02 (1.01–1.03)	0.002	1.02 (1.01–1.03)	0.007
Race/ethnicity				
Non-Hispanic White	Ref		Ref	
Non-Hispanic Black	1.53 (1.39–1.68)	<0.0001	1.28 (1.16–1.42)	<0.0001
Hispanic	1.17 (1.07–1.27)	0.001	0.96 (0.88–1.06)	0.42
Asian	0.85 (0.75–0.97)	0.014	0.83(0.73–0.95)	0.001
Other/unknown	0.88 (0.62–1.24)	0.46	0.78 (0.55–1.11)	0.17
Insurance				
Managed care	Ref		Ref	
Medicare	1.33 (1.21–1.46)	<0.0001	1.20 (1.08–1.34)	0.0001
Medicaid	1.78 (1.61–1.96)	<0.0001	1.29 (1.16–1.43)	<0.0001
Other insurance	0.91 (0.82–1.01)	0.07	0.94 (0.85–1.04)	0.25
Not insured or unknown	1.31 (1.10–1.57)	0.002	1.17 (0.98–1.40)	0.08
Socioeconomic status (SES)				
Highest SES	Ref		Ref	
Lowest SES	1.62 (1.45–1.82)	<0.0001	1.20 (1.06–1.36)	0.004
Lower-middle SES	1.44 (1.29–1.61)	<0.0001	1.19 (1.06–1.33)	0.004
Middle SES	1.36 (1.22–1.52)	<0.0001	1.21 (1.08–1.35)	0.001
Higher-middle SES	1.17 (1.05–1.31)	0.005	1.06 (0.95–1.18)	0.33
Marital status				
Not married			Ref	
Married	0.77 (0.72–0.83)	<0.0001	0.94 (0.88–1.01)	0.10
Tumor stage				
I	Ref		Ref	
II	2.83 (2.52–3.18)	<0.0001	2.59 (2.30–2.92)	<0.0001
III	9.03 (8.02–10.18)	<0.0001	8.28 (7.34–9.35)	<0.0001
IV	37.09 (32.37–42.50)	<0.0001	37.82 (32.74–43.68)	<0.0001
Received NCCN-adherent care				
Yes			Ref	
No	1.15 (1.07–1.23)	0.0002	1.21 (1.11–1.31)	<0.0001

## Data Availability

This study used data from the California Cancer Registry, which is publicly available at https://www.ccrcal.org/retrieve-data (accessed on 15 October 2023).

## Supplementary Materials

The following supporting information can be downloaded at https://www.mdpi.com/article/10.3390/cancers15235586/s1. Figure S1: Kaplan–Meier disease-specific survival graph with log-rank test by treatment adherence status for stages I, II, III, and IV, respectively; Table S1: Algorithm defining NCCN-adherent treatment for TNBC; Table S2: Distribution of patient characteristics by race/ethnicity.
