# Supplementary material for: Disparities in Receipt of National Comprehensive Cancer Network Guideline-Adherent Care and Outcomes among Women with Triple-Negative Breast Cancer by Race/Ethnicity, Socioeconomic Status, and Insurance Type
Source: Cancers (Basel). 2023 Nov 26;15(23):5586. doi: 10.3390/cancers15235586 (PMC10705726; doi:10.3390/cancers15235586)
Supplement: Supplementary file 1 [file cancers-15-05586-s001.zip › cancers-2691717-supplementary.pdf]

Table S1. Algorithm defining NCCN-adherent treatment for TNBC [16].

Tumor Characteristics		Adherent Treatment			
Lymph Node	**Tumor Size**	**Surgery and Radiation Therapy**	**Sentinel Lymph Node Surgery**	**Axillary Lymph Node Surgery**	**Chemotherapy**
		Stage I, II, IIIA with N1			
N0	<=10 mm	Total mastectomy or BCS +radiation	Yes		
N0	>10 mm	Total mastectomy or BCS + radiation	Yes		Yes
N1mi	<=10 mm	Total mastectomy or BCS + radiation	Yes		
N1mi	>10 mm	Total mastectomy or BCS + radiation	Yes		Yes
Positive	Any	Total mastectomy or BCS + radiation	Yes		Yes
		Stage IIIA with N2+, IIIB, IIIC			
Any	Any	Total mastectomy + radiation or BCS + radiation		Yes	Yes
		Stage IV			
Any	Any				Yes

Abbreviations: BCS, breast-conserving surgery; mi, micro-metastasis.

Table S2. Distribution of patient characteristics by race/ethnicity.

	Non-Hispanic White		Non-Hispanic Black		Hispanic		Asian		P-value [a]
	N	(%)	N	(%)	N	(%)	N	(%)	
	8930	53.6	2085	12.5	3880	23.3	1752	10.5	
Age at diagnosis (years)									<0.0001
18-44	1424	15.9	382	18.3	1243	32.0	428	24.4	
45-54	2177	24.4	627	30.1	1214	31.3	499	28.5	
55-64	2643	29.6	604	29.0	843	21.7	439	25.1	
65-79	2686	30.1	472	22.6	580	14.9	386	22.0	
Year of Diagnosis									<0.0001
2004-2009	5595	62.7	1267	60.8	2208	56.9	1010	57.6	
2010+	3335	37.3	818	39.2	1672	43.1	742	42.4	
Insurance									<0.0001
Manage Care	4564	51.1	1194	57.3	1829	47.1	921	52.6	
Medicare	1664	18.6	358	17.2	410	10.6	223	12.7	

Medicaid	544	6.1	274	13.1	1009	26.0	245	14.0	
Other Insurance	1856	20.8	186	8.9	485	12.5	311	17.8	
Not insured or unknown	302	3.4	73	3.5	147	3.8	52	3.0	
Neighborhood socioeconomic status. (SES)									<0.0001
Lowest SES	692	7.7	529	25.4	1193	30.7	144	8.2	
Lower-middle SES	1341	15.0	537	25.8	1008	26.0	264	15.1	
Middle SES	1874	21.0	465	22.3	777	20.0	334	19.1	
Higher-middle SES	2356	26.4	375	18.0	574	14.8	457	26.1	
Highest SES	2667	29.9	179	8.6	328	8.5	553	31.6	
Marital status									<0.0001
Not married	3481	39.0	1300	62.4	1610	41.5	538	30.7	
Married	5449	61.0	785	37.6	2270	58.5	1214	69.3	
Tumor stage									<0.0001
I	3377	37.8	614	29.4	1092	28.1	588	33.6	
II	3961	44.4	970	46.5	1914	49.3	859	49.0	
III	1264	14.2	379	18.2	714	18.4	245	14.0	
IV	328	3.7	122	5.9	160	4.1	60	3.4	
Received adherent care									<0.0001
No	5893	66.0	1480	71.0	2705	69.7	1179	67.3	
Yes	3037	34.0	605	29.0	1175	30.3	573	32.7	

(A) Stage I

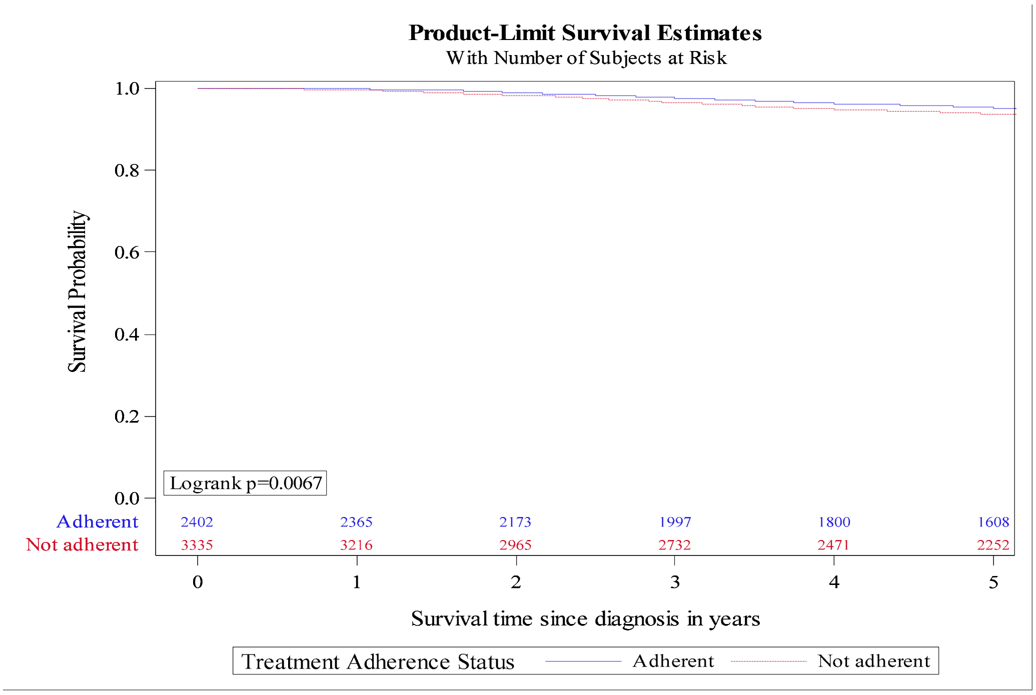

(B) Stage II

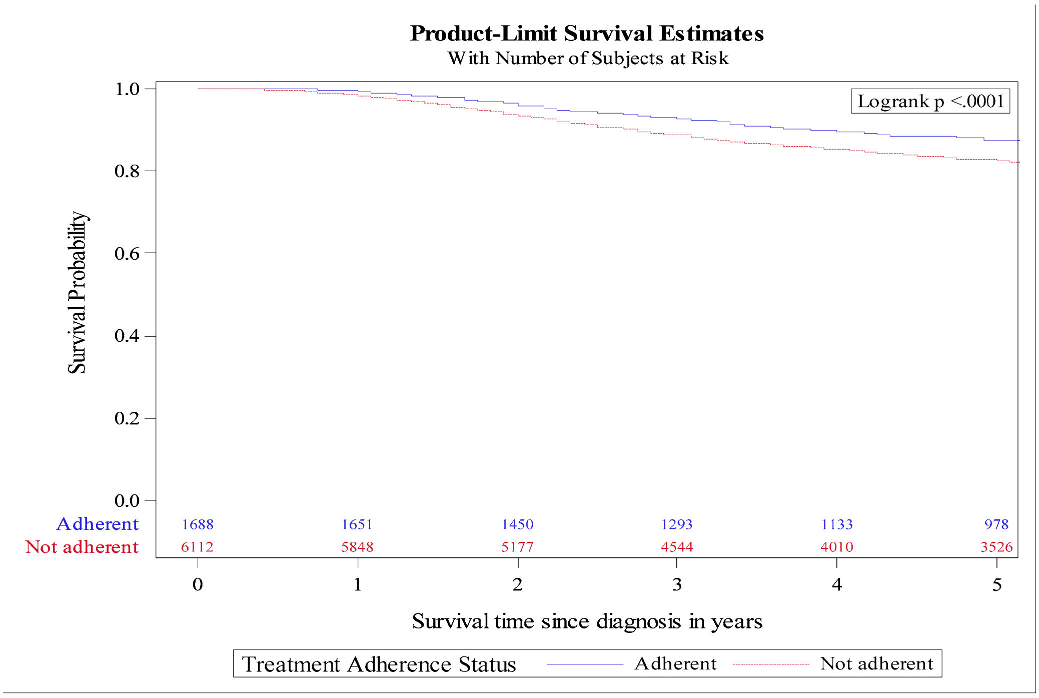

(C) Stage III

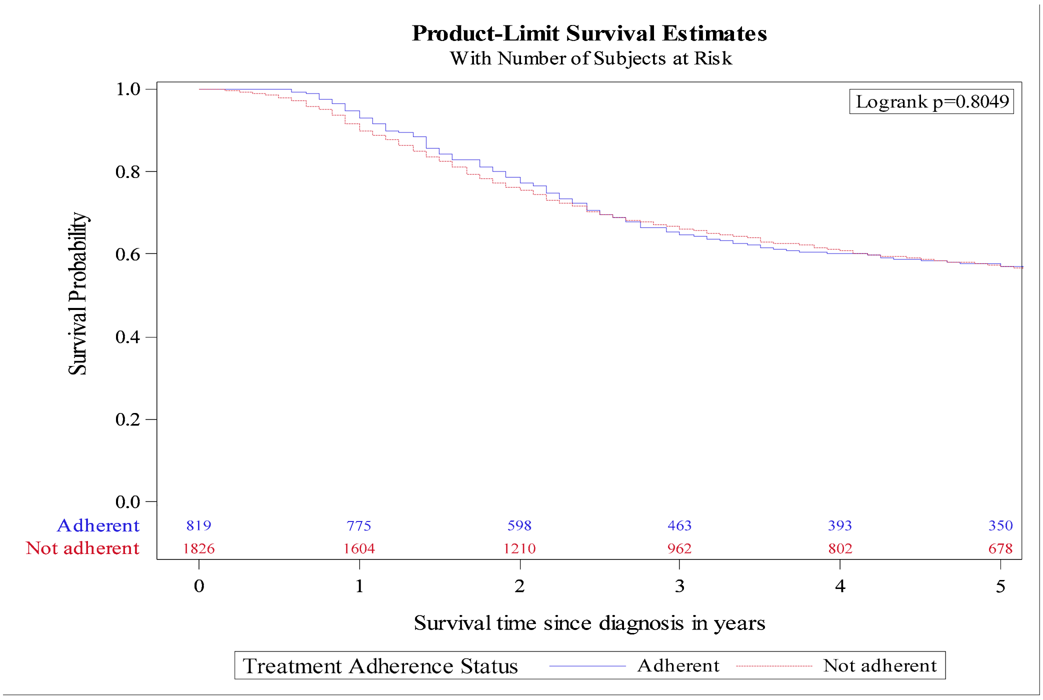

(D) Stage IV

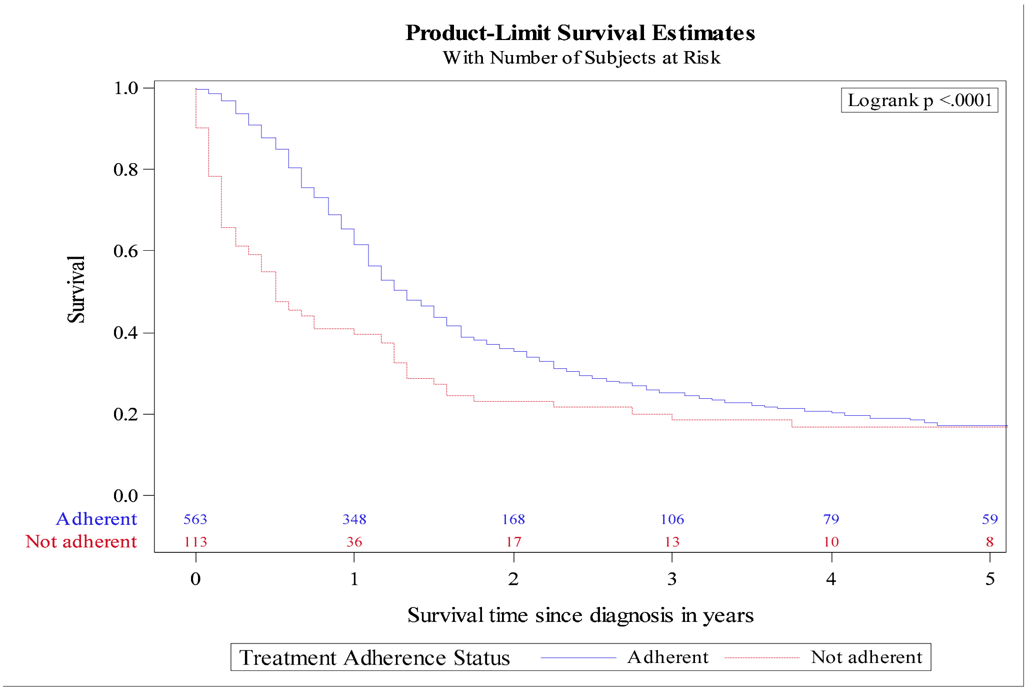

Figure S1. Kaplan-Meier disease-specific survival graph by treatment adherent status.
